# Physics-Informed Tensor-Train ConvLSTM for Volumetric Velocity Forecasting of the Loop Current

**DOI:** 10.3389/frai.2021.780271

**Published:** 2021-12-24

**Authors:** Yu Huang, Yufei Tang, Hanqi Zhuang, James VanZwieten, Laurent Cherubin

**Affiliations:** ^1^Department of Electrical Engineering and Computer Science, Florida Atlantic University, Boca Raton, FL, United States; ^2^Department of Civil, Environmental and Geomatics Engineering, Florida Atlantic University, Boca Raton, FL, United States; ^3^Ocean Dynamics and Modeling, Harbor Branch Oceanographic Institute, Florida Atlantic University, Fort Pierce, FL, United States

**Keywords:** physics-informed deep learning, time series forecasting, spatiotemporal predictive modeling, loop current, ocean current modeling, volumetric velocity prediction

## Abstract

According to the National Academies, a week long forecast of velocity, vertical structure, and duration of the Loop Current (LC) and its eddies at a given location is a critical step toward understanding their effects on the gulf ecosystems as well as toward anticipating and mitigating the outcomes of anthropogenic and natural disasters in the Gulf of Mexico (GoM). However, creating such a forecast has remained a challenging problem since LC behavior is dominated by dynamic processes across multiple time and spatial scales not resolved at once by conventional numerical models. In this paper, building on the foundation of spatiotemporal predictive learning in video prediction, we develop a physics informed deep learning based prediction model called—Physics-informed Tensor-train ConvLSTM (PITT-ConvLSTM)—for forecasting 3D geo-spatiotemporal sequences. Specifically, we propose (1) a novel 4D higher-order recurrent neural network with empirical orthogonal function analysis to capture the hidden uncorrelated patterns of each hierarchy, (2) a convolutional tensor-train decomposition to capture higher-order space-time correlations, and (3) a mechanism that incorporates prior physics from domain experts by informing the learning in latent space. The advantage of our proposed approach is clear: constrained by the law of physics, the prediction model simultaneously learns good representations for frame dependencies (both short-term and long-term high-level dependency) and inter-hierarchical relations within each time frame. Experiments on geo-spatiotemporal data collected from the GoM demonstrate that the PITT-ConvLSTM model can successfully forecast the volumetric velocity of the LC and its eddies for a period greater than 1 week.

## 1. Introduction

As part of the North Atlantic western boundary current system, the Loop Current (LC) originates as the Yucatan Current, flows northward into the Gulf of Mexico (GoM), and veers east to exit through the Straits of Florida where it becomes the Florida Current, which then transitions into the Gulf Stream system. The LC dynamics is characterized by the shedding of clockwise (anticyclonic) rotating eddies that move westward further into the GoM. The LC and its eddies together are called the Loop Current System (LCS). On April 20, 2010, the oil drilling rig Deepwater Horizon, operating in the Macondo Prospect in the GoM, exploded and sank, resulting in 4 million barrels of oil gushing uncontrolled into the Gulf over an 87-day period, before it was finally capped on July 15, 2010 (The Commission, 2011). This disaster, also known as the GoM Oil Spill, threatened livelihoods, precious habitats, and even a unique way of life in the GoM region. Since the disaster, a large amount of research reported in the literature has improved scientific understanding and forecasting of the LC with aspirations for alleviating impacts on ecosystems and for future oil-spill prevention. The ability to predict the LC evolution is critical and fundamental to almost all aspects of the GoM community, including (1) anthropogenic and natural disaster response, (2) the prediction of short-term weather anomalies, and hurricane intensity and trajectories, (3) national security and safety, and (4) ecosystem services (Walker et al., 2009). Due to a reinforcing interaction between seasonal hurricanes and the LCS, long-term prediction of the LCS states is of particular interest and becoming increasingly relevant for mitigating potential environmental and ecological threats.

Modeling and forecasting the dynamics of the LC in the GoM remains an open scientific problem. The National Academies of Science, Engineering and Medicine (NASEM) published a report (National Academies Press, 2018) specifically calling for the development of models that are capable of forecasting (a) current speed, vertical structure, and duration of the LC and its eddies at a given location one week in advance, (b) LC propagation one month ahead, and (c) eddy shedding events up to 13 weeks ahead. Current prediction models fall mostly into one category which consists of numerical model-based methods. They primarily use finite-differences or finite-element techniques to discretize partial differential equations (PDEs) of momentum and tracer transport, and conservation. Although the various models used differ in their details, features such as the LC eddy shedding periods, eddy propagation, flow transport, and vertical structure in the LC and the formation of LC frontal cyclonic eddies are relatively similar. Some of these models use data assimilation techniques to constrain their model solution with remote sensing and *in-situ* observations (see Wang et al., 2019, 2021 for a literature review of the models). However, despite the data constraint the reliable prediction window for surface and subsurface currents of the LCS is from a few hours to 2 days at most (Wang et al., 2021).

Therefore, the long-term forecasting of the volumetric velocity has remained an unattainable goal despite the large amount of research focused on the LC dynamics. The major challenges of the LC velocity field prediction are found in the nature of the LC current dynamics which is dominated by instabilities and multi-scale interactions in the temporal, spatial and frequency domain (Yang et al., 2020). These interactions occur not only in the horizontal but also in the vertical dimension, and are controlled by the vertical discretization scheme chosen in the numerical ocean model. The choice of the vertical coordinate system has a significant impact of the physics and processes resolved by the model, which results in diverging solution between models, among other factors (Halliwell, 2004; Bruciaferri et al., 2018).

In this paper, we develop a novel technique to predict the evolution of this type of geophysical dynamical system, named the Physics-informed Tensor-train ConvLSTM (PITT-ConvLSTM) model, that enables 4D spatiotemporal LC prediction, namely the prediction of a continuous sequence of 3D flow fields (see section 4.1). We address the problem of modeling the LCS based on convolutional Long Short Term Memory (LSTM) networks (ConvLSTM) by incorporating prior domain knowledge described as non-linear differential dynamic equations. Our PITT-ConvLSTM model can leverage required physics explicitly, and learn implicit patterns from data. Moreover, unlike most of the first-order ConvLSTM-based approaches, we propose a higher-order generalization to ConvLSTM with convolutional tensor-train decomposition (Su et al., 2020) to effectively learn the long-term spatiotemporal structure in the LCS.

The remainder of this paper is organized as follow. In section 2, we discuss the motivation of proposing physics informed neural network for LC forecasting. Section 3 presents a gallery of related works. Section 4 describes the dataset and its pre-processing method. Section 5 describes the proposed framework and our main results including the baselines are presented in section 6. Finally, concluding remarks are made in section 7.

## 2. Motivation

In the past decades, tremendous progress has been made in understanding multiscale physics in diverse geophysics applications, by numerically solving partial differential equations (PDEs) using finite differences, finite elements, spectral, etc. Such numerical model-based approaches still cannot seamlessly incorporate noisy data into existing algorithms, mesh generation remains complex, and high-dimensional problems governed by parameterized PDEs cannot be tackled (Karniadakis et al., 2021), showing limitation in solving real-world physical problem. Machine learning has emerged as a promising alternative. They are purposed to discover complex patterns from large volumes of data. However, training deep neural networks requires big data, while the volume of useful experimental/real-world data for complex physical systems is limited. For instance, in our case, gaps remain in oceanographic observations necessary to further understanding and prediction of the LCS, as the volumetric velocity data of the LCS is inadequate compare to other data domains (Hamilton et al., 2016).

Most machine-learning-based approaches are currently unable to extract interpretable information and knowledge from data. Moreover, machine-learning-based approaches, as a purely data-driven method in nature, are easy to be overfitting. Predictions may be physically inconsistent or implausible, owing to extrapolation or observational biases that may lead to poor generalization performance (Karniadakis et al., 2021). While numerical model-based approaches are generally limited by their high complexity and incompleteness, they do not require large amounts of data and retain interpretation. To this end, incorporating prior physics knowledge into machine learning can help enhance generalization and interpretability, and reduce the need for massive amounts of training data. In general, physics can be used as “teachers” to guide the discovery of meaningful and explainable machine learning models. It is hypothesized that the combination of machine learning and physics could lead to performance gains by leveraging the advantages of both sides.

Machine learning models can be trained from additional information obtained by enforcing the law of physics (Raissi et al., 2019). By doing so, such physics-informed neural networks can (1) provide theoretical guidance that the model is required to follow and thus, facilitate the model training with fewer labeled data; and (2) improve the model robustness for unseen data and prevent over-fitting. Considering the difficulty in identifying which observations are following the physics explicitly and how much in accordance to it, the law of physics will not be directly applied to the raw observations, but rather to the latent space for representation learning (e.g., the hidden states of the deep neural networks). Therefore, the neural networks would remain flexible to extract unknown patterns as well, instead of being strictly constrained to the knowledge of physics.

## 3. Related Work

### 3.1. Physics-Informed Neural Networks

Physics is one of the fundamental pillars that can explain how nature behaves. Because it is unable to describe all the rules governing real-world events, machine learning is desired to bridge the known physics and real-world observations. Physics-informed machine learning integrates data and mathematical models, even in partially understood, uncertain, and high-dimensional contexts (Karniadakis et al., 2021). In addition, (Raissi et al., 2017; Raissi, 2018; Raissi and Karniadakis, 2018) demonstrated that neural networks are capable of finding solutions to partial differential equations (PDEs), which enables us to obtain fully differentiable physics-informed models with respect to all input coordinates and free parameters. (de Bezenac et al., 2019) demonstrated how fluid physics could be incorporated for forecasting sea surface temperature, and such a method not only captures the dominant physics but also infers unknown patterns by CNNs. Further, a number of physics-informed models were developed, assuming that neural networks can learn complex dynamic interactions and simulate unseen dynamics based on current states (Kipf et al., 2018; Sanchez-Gonzalez et al., 2018). Unlike those works that implicitly extract latent patterns from data only, the physics-informed graph networks proposed in Seo and Liu (2019) allow incorporating known physics and simultaneously extracting latent patterns in data that is unable to be captured by numerical model-based approaches.

### 3.2. Recurrent Neural Network With Tensor-Train Decomposition

Tensor-train decomposition (TTD) factorizes model parameters into smaller tensors (Oseledets, 2011) in order to reduce the space dimension. This decomposition is especially beneficial in multi-relational data analysis, and has been widely used in machine learning, including CNNs (Su et al., 2018, 2020; Kolbeinsson et al., 2019), RNNs (Yang et al., 2017; Su et al., 2020), and transformers (Ma et al., 2019). A multiplicative RNN with factorized (weights) tensors was proposed for input-state interactions (Sutskever et al., 2011). Further, (Yang et al., 2017) factorized the input-to-hidden weights within each cell by TTD, and showed improvement in video classification. Different from the first-order RNNs mentioned above, higher-order RNNs (Soltani and Jiang, 2016) require many more parameters since they introduce connections across multiple previous time steps for better long-term dynamics learning. This was improved by TTD-RNNs (Yu et al., 2018), whose higher-order structures within each cell were compressed by TTD, which was shown to improve the model performance in video classification. Recently, (Su et al., 2020) introduced a new convolutional TTD and constructed Convolutional Tensor-Train LSTM that was able to capture higher-order spatiotemporal correlations.

## 4. Dataset

### 4.1. Data Description

**Figure 1:**
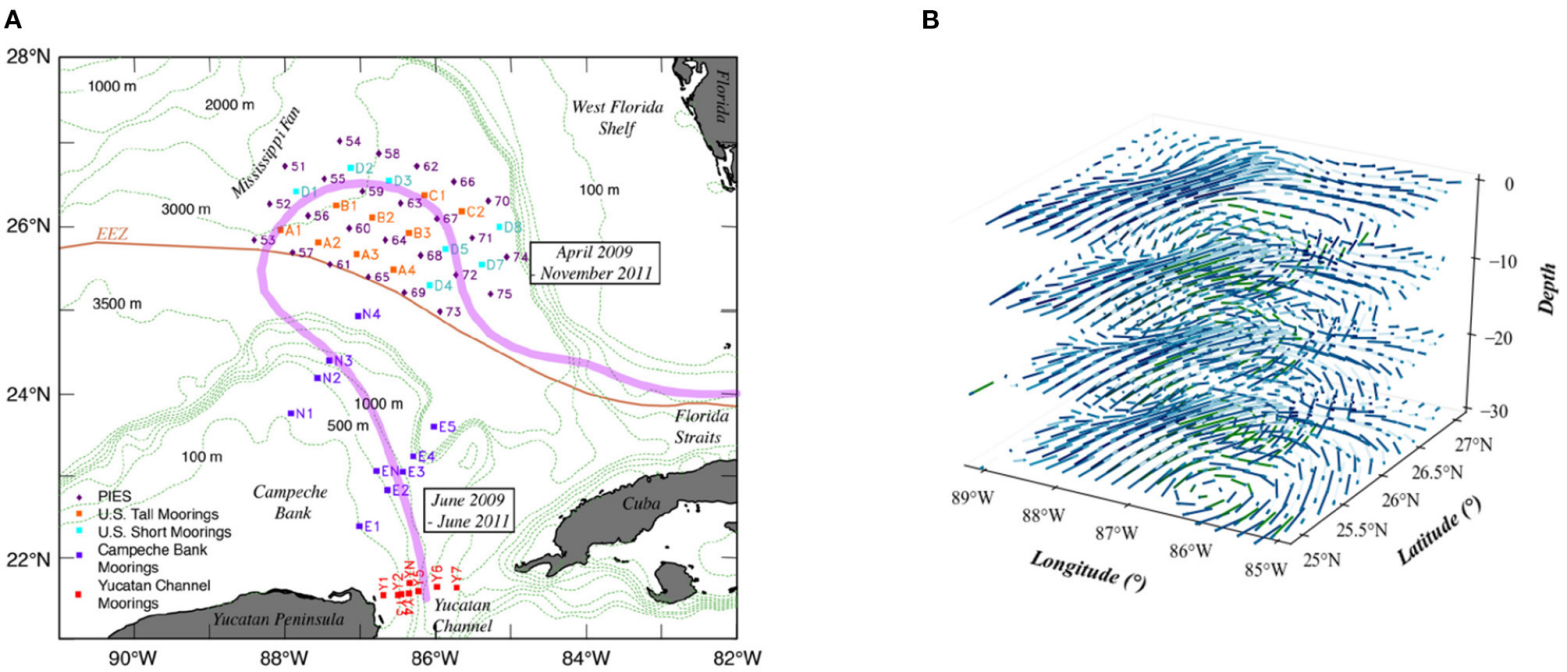
**(A)** Locations of moorings and PIES deployed in the U.S. and Mexican sectors in the eastern Gulf of Mexico (Hamilton et al., 2016). **(B)** Visualization of a time slice $X_{t}$ at 10 (×20 m) depth intervals.

The dataset used in this study was produced by a funded observational program called "Dynamics of the Loop Current in US Waters Study" (Hamilton et al., 2016) and consisted of a high-density array of moored instruments over a two-and-a-half-year period, beginning in April 2009. The observational array (Figure 1A) collected LC velocity and density structure over the region where the LC extended northward and, more importantly, where eddy shedding events occurred most often. This sensor array consisted of nine tall-moorings, seven short-moorings, 25 pressure-equipped inverted echo sounders (PIES), and remote sensors. Each of the nine tall-moorings recorded water velocities using an upward facing 75 kHz acoustic Doppler current profiler (ADCP) deployed at an approximate depth of 450 m. Additionally, point current meters were attached to each tall mooring at five depths ranging from around 600 to 3,000 m. Collectively, these instruments provided high resolution water velocity data from about 60 to 440 m depths and at much lower resolution below this. Each of the seven short-moorings provided near bottom water velocity data using a single point current sensor deployed 100 m above the sea floor. PIES sensors measured both pressure and acoustic round trip travel times from the sea floor to the sea surface. Vertical profiles of density, salinity, and temperature were created from these data. The PIES pressure records were also combined with estimated horizontal density gradients to calculate geostrophic water velocities. The complete procedure for producing these mapped velocity fields was described in Donohue et al. (2016b) and Hamilton et al. (2016).

The utilized dataset, summarized in Table 1, contains velocity data gathered from April 2009 to June 2011. The southwest edge of this array was just to the US side of the exclusive economic zone (EEZ) from 25°50.2′N; 88°24.5′W to 24°59.2′N; 85°56.8′W, with the northeast edge of this array ~160 km to the US side of the EEZ. The horizontal separation between moorings was around 50−80 km and between the PIES sensors was around 40−50 km. Sampling frequency from the multiple sensors varied from minutes to hours. In this study, we utilize a time series which was processed with a fourth order Butterworth filter and sub-sampled at 12-h intervals. Consequently, the dataset contains a total of 1,810 records, covering 905 days. At each time step *t*, as shown in Figure 1B, the data $X_{t}$ were formatted as $X_{t}\subseteq {\mathbb{R}}^{D\times H\times W\times C}$, where each dimension represents *Depth* (30), *Longitude* (29), *Latitude* (36), and *Channel* (2), respectively. *Channel* stands for the geostrophic velocity vector (*u, v*). The first 80% (June 2009 to December 2010) of the dataset was reserved for training and the remaining 20% (January 2011 to June 2011) for prediction and validation (Shi et al., 2010).

**Table 1:** Statistics of ocean current dataset.

**Time range**	**Sampling rate**	**Spatial range**	**Dimensions**				
			**Depth**	**Length**	**Width**	**Channel**	**Steps (T)**
April 2009 to June 2011	12 h	25°50.2′*N*, 88°24.5′*W* to 24°59.2′*N*, 85°56.8′*W*	30 (D)	29 (H)	36 (W)	2 (C)	1810 (T)

### 4.2. Data Preprocessing

Empirical Orthogonal Function (EOF) analysis has been extensively used in oceanic and atmospheric sciences. In addition to its ability to decompose a time series into its temporal and spatial components, EOF can drastically reduce the dimension while preserving data integrity. Considering the non-linear dynamics and high dimension characteristics of oceanic phenomena in this work, the EOF analysis is employed to represent spatial patterns (*EOFs*) and temporal components (Principal Components, *PCs*) of the LCS in the GoM region. Specifically, the EOF analysis was conducted to extract the *PCs* of zonal and meridional velocity of ocean current. The *PCs* may contain otherwise hidden and medium-term uncorrelated patterns (Navarra and Simoncini, 2010). Further, *PCs* are used to train the PITT-ConvLSTM prediction model, with details given in section 5.

Singular Value Decomposition (SVD) is a mathematical tool to determine both the *EOFs* and *PCs* simultaneously. For the convenience of ocean modeling, the ocean is sliced into layers in the depth direction. Here, SVD is applied to each depth slice S to obtain two unitary orthogonal matrices (*U* and *V*) and one diagonal eigenvalue matrix Σ. Consequently, *U*Σ represents the temporal *PCs* and *V*^*T*^ the spatial *EOFs*. Thus, S can be represented as:


(1) $$\begin{array}{l} S=U\Sigma V^{T}\\ \;↔\underset{S}{\underset{︸}{\left[\begin{array}{ccc} s_{1,1} & \cdots & s_{1,m}\\ \vdots & \ddots & \vdots \\ s_{n,1} & \cdots & s_{n,m} \end{array}\right]}}=\underset{PCs}{\underset{︸}{\left[\begin{array}{ccc} p_{1,1} & \cdots & p_{1,m}\\ \vdots & \ddots & \vdots \\ p_{n,1} & \cdots & p_{n,m} \end{array}\right]}}\underset{EOFs}{\underset{︸}{\left[\begin{array}{ccc} e_{1,1} & \cdots & e_{1,m}\\ \vdots & \ddots & \vdots \\ e_{m,1} & \cdots & e_{m,m} \end{array}\right]}} \end{array}$$

After the EOF analysis, we concatenate the flattened *PCs* along the depth dimension. In this way, the raw data $X\subseteq {\mathbb{R}}^{T\times D\times H\times W\times C}$ is compressed to $X^{\prime }\subseteq {\mathbb{R}}^{T\times D\times P\times C}$, where *P* is the number of selected elements in *PCs* (we utilized the first 50 PCs that contained over 95% of the energy). The 3D volume sequence prediction ${\left\{X^{\left(t\right)}\right\}}_{t=1}^{T}$, in consequence, is compressed to a 2D matrix sequence prediction ${\left\{X^{\prime (t)}\right\}}_{t=1}^{T}$. Each row of the 2D matrix $X^{\prime (t)}\left(i,:\right)$ (flattened PCs) represents a depth slice of the original 3D volume $X^{\left(t\right)}\left(i,:,:\right)$. The model in section 5 will be trained to predict future *PCs*, which could also be used to reconstruct predicted 3D volume by simply multiplying them by the known *EOFs* (constant) as shown in Equation (1).

## 5. Method

### 5.1. Method Overview

Convolutional LSTM network (ConvLSTM), a basic building block for sequence-to-sequence prediction, demonstrated promising performance in video forecasting. In ConvLSTM, the spatial information is encoded explicitly as tensors in the LSTM cells, where each cell is a first-order Markovian model (i.e., the hidden states are updated based on their adjacent step). Since ConvLSTM is successful in modeling complex behaviors and extracting abstract features through real-world data (Xingjian et al., 2015), it is natural to explore how such a predictive model can be used to solve practical problems in geo-spatiotemporal modeling domains with higher-order dynamics.

**Figure 2:**
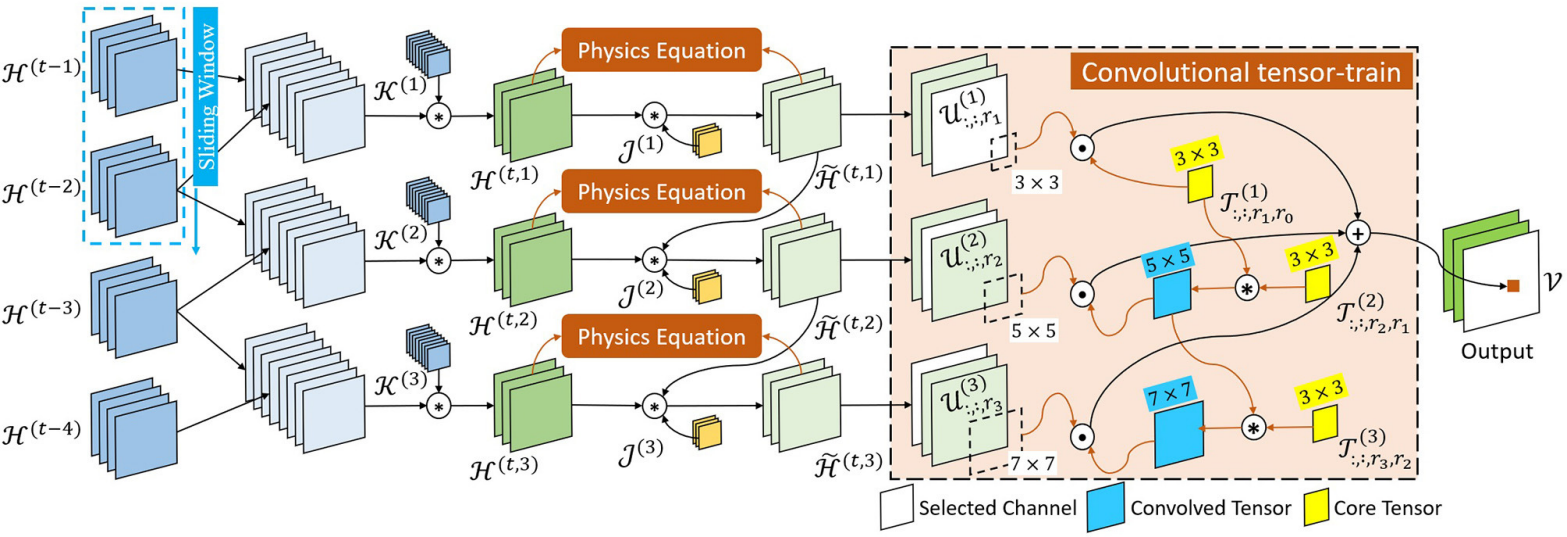
Schematic of φ{T,H} within the PITT-ConvLSTM cell.

**Figure 3:**
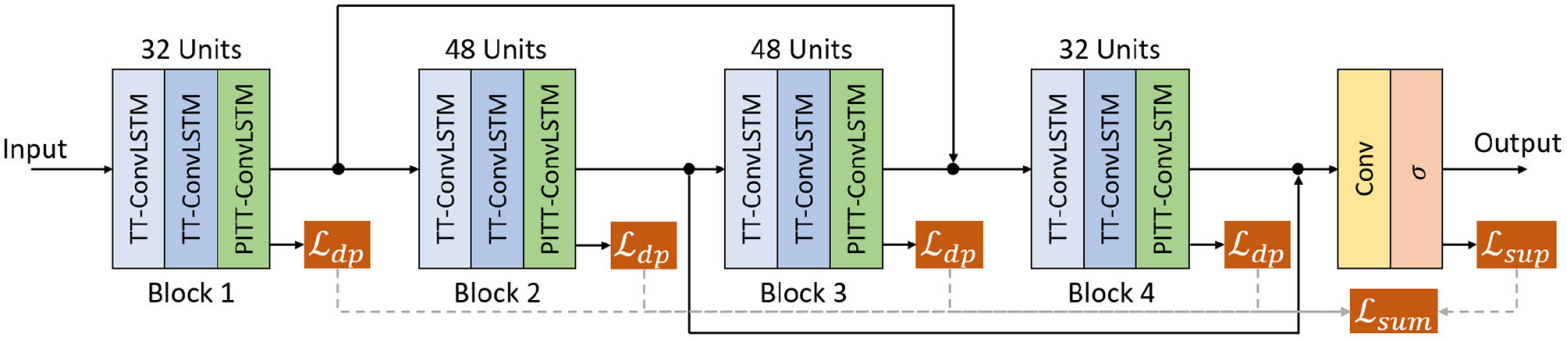
Illustration of the recurrent network architecture of 12-layer.

In this paper, we propose a novel model Physics-informed Tensor-train ConvLSTM (PITT-ConvLSTM), a ConvLSTM network under physics constraints. The ConvLSTM is integrated with convolutional tensor-train to model higher-order spatiotemporal correlations explicitly, with its hidden states incorporated with prior physics. The proposed model is illustrated in Figures 2, 3.

### 5.2. ConvLSTM With Convolutional Tensor-Train Decomposition

Different from the conventional LSTM, the ConvLSTM introduces convolution tensor operation into input-to-state and state-to-state transitions within each recurrent cell (Xingjian et al., 2015). In ConvLSTM, all features are encoded as third-order tensors with dimensions (*H* × *W* × *C*). At each time step *t*, a ConvLSTM cell updates its hidden states $H^{\left(t\right)}$ based on the previous $H^{\left(t-1\right)}$ and the current input $X^{\left(t\right)}$:


(2) $$\begin{array}{l} \left[I^{\left(t\right)},F^{\left(t\right)},{\tilde{C}}^{\left(t\right)},O^{\left(t\right)}\right]=\sigma \left(W_{input}*X^{\left(t\right)}+T_{hidden}*H^{\left(t-1\right)}\right) \end{array}$$

where σ(·) applies sigmoid on the input gate $I^{\left(t\right)}$, forget gate $F^{\left(t\right)}$, and output gate $O^{\left(t\right)}$, and *tanh*(·) on memory cell ${\tilde{C}}^{\left(t\right)}$. The $I^{\left(t\right)},F^{\left(t\right)},O^{\left(t\right)},{\tilde{C}}^{\left(t\right)}\subseteq {\mathbb{R}}^{H\times W\times C}$, and the parameters are characterized by two 4*th* order tensors $W_{input}\subseteq {\mathbb{R}}^{K\times K\times S\times 4C}$ and $T_{hidden}\subseteq {\mathbb{R}}^{K\times K\times C\times 4C}$, where *K* is the kernel size, and *S* and *C* are the numbers of channels of X and H separately.

To capture multi-steps spatiotemporal correlations in ConvLSTM, we introduce a higher-order recurrent unit, where the hidden state $H^{\left(t\right)}$ is updated based on the current input $X^{\left(t\right)}$ and its *n* previous steps ${\left\{H^{\left(t-l\right)}\right\}}_{l=1}^{n}$ with an *m*-order convolutional tensor-train decomposition $\varphi \left({\left\{T^{\left(l\right)}\right\}}_{l=1}^{m},\cdot \right)$ as follows:


(3) $$\begin{array}{l} \left\{{\tilde{H}}^{\left(t,o\right)}\right\}=f\left(J^{\left(o\right)},K^{\left(o\right)},{\left\{H^{\left(t-l\right)}\right\}}_{l=1}^{n}\right),\forall o\subseteq \left[m\right] \end{array}$$

(4) $$\begin{array}{l} \left[I^{\left(t\right)},F^{\left(t\right)},{\tilde{C}}^{\left(t\right)},O^{\left(t\right)}\right]=\sigma (W_{input}*X^{\left(t\right)}+\varphi \{{\left\{T^{\left(o\right)}\right\}}_{o=1}^{m},\\ \;{\left\{{\tilde{H}}^{\left(t,o\right)}\right\}}_{o=1}^{m}\}) \end{array}$$

where the *m*-order convolutional tensor-train $\varphi \left({\left\{T^{\left(l\right)}\right\}}_{l=1}^{m},\cdot \right)$ are parameterized by *m* core tensors ${\left\{T^{\left(l\right)}\right\}}_{l=1}^{m}$. Considering the consistency constraint, the previous *n* steps are mapped into *m* intermediate tensors ${\tilde{H}}^{\left(t,o\right)}$ by f(J,K,·) in Equation (3) at first, where K and J are 3D convolutional kernels. Note that f(J,K,·) is a mapping function with dynamic physics constraints, which will be discussed in section 5.3.

**Figure 4:**
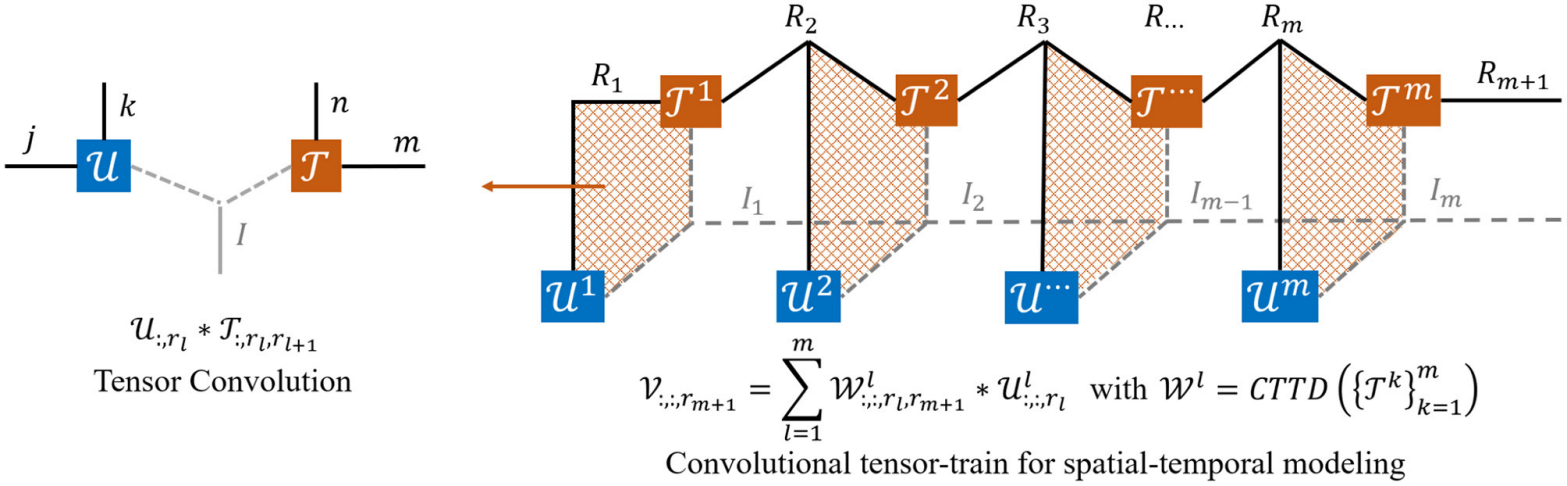
The tensor diagrams of convolutional tensor-train (Su et al., 2020) for spatial-temporal modeling. $U^{1}$ is first interacted with $T^{1}$, then the resulting intermediate tensor is further interacted with $T^{2}$ and $U^{2}$. The process repeats until all tensors are merged into one.

The convolutional tensor-train decomposition, first proposed by Su et al. (2020), is a counterpart of tensor-train decomposition (TTD) which aims to represent a higher-order tensor $T\subseteq {\mathbb{R}}^{I_{1}\times \cdots \times I_{m}}$ in a set of smaller and lower-order core tensors ${\left\{T^{\left(l\right)}\right\}}_{l=1}^{m}$ with $T^{\left(l\right)}\subseteq {\mathbb{R}}^{I_{l}\times R_{l}\times R_{l-1}}$. The ranks ${\left\{R^{\left(l\right)}\right\}}_{l=1}^{m}$ here control the number of parameters in the tensor-train format. In this way, the original T of size $\prod_{l=1}^{m}I_{l}$ is compressed to $\overset{m}{\underset{l=1}{\sum }}I_{l}R_{l-1}R_{l}$, i.e., the complexity only grows linearly with the order *m* (assuming *R*_*l*_'s are constants). Similar to TTD, φ is designed to significantly reduce both parameters and operations of higher-order spatio-temporal recurrent models by factorizing a large convolutional kernel into a chain of smaller kernels. The details of convolutional tensor-train decomposition $T=CTTD{\left\{T^{\left(l\right)}\right\}}_{l=1}^{m}$ is formulated as:


(5) $$\begin{array}{l} T_{:,r_{1},r_{m+1}}≜\overset{R_{2}}{\underset{r_{2}=1}{\sum }}\cdots \overset{R_{m}}{\underset{r_{m}=1}{\sum }}T_{:,r_{1},r_{2}}*\cdots *T_{:,r_{m},r_{m+1}}^{m} \end{array}$$

and the convolutional tensor-train for spatiotemporal modeling (Figure 4) can be equivalently stated as:


(6) $$\begin{array}{l} V_{:,r_{l+1}}^{l+1}=\overset{R_{l}}{\underset{r_{l}=1}{\sum }}T_{:,r_{l},r_{l+1}}^{l}*\left(V_{:,r_{l}}^{l}+U_{:,r_{l}}^{l}\right) \end{array}$$

where U is the input feature corresponding to $\tilde{H}$. ${\left\{V^{l}\right\}}_{l=1}^{m}$ are intermediate results, in which $V^{l}\subseteq {\mathbb{R}}^{H\times W\times R_{l}}$ for *l* > 1, and $V^{1}\subseteq {\mathbb{R}}^{H\times W\times R_{m}}$ is initialized as all zeros and final prediction is returned as $V=V^{m+1}$.

**Proof of Equation (6)** The concrete mathematical expression of convolutional tensor-train decomposition $CTTD{\left\{T^{\left(l\right)}\right\}}_{l=1}^{m}$ is formulated in Equation (5) and illustrated in Figure 4. Consider a model that predicts a feature $V\in {\mathbb{R}}^{I_{m}\times R_{m+1}}$ based on inputs U. *R*_*l*_ is the number of channel in $U^{l}$. The proof is inspired by the work of Su et al. (2020). Since $V^{1}$ is initialized as an all zero tensor, the Equation (6) can be rewritten as:


(7a) $$\begin{array}{l} V_{:,r_{m}+1} \end{array}$$

(7b) $$\begin{array}{l} =\overset{m}{\underset{l=1}{\sum }}\overset{R_{l}}{\underset{r_{l}=1}{\sum }}CTTD{\left({\left\{T\right\}}_{t=l}^{m}\right)}_{:,r_{l},r_{m+1}}*U_{:,r_{l}}^{l}\\ +\overset{R_{1}}{\underset{r_{l}=1}{\sum }}CTTD{\left({\left\{T\right\}}_{t=1}^{m}\right)}_{:,r_{1},r_{m+1}}*V_{:,r_{1}}^{1} \end{array}$$

(7c) $$\begin{array}{l} =\overset{m}{\underset{l=2}{\sum }}\overset{R_{l}}{\underset{r_{l}=1}{\sum }}CTTD{\left({\left\{T\right\}}_{t=l}^{m}\right)}_{:,r_{l},r_{m+1}}*U_{:,r_{l}}^{l}\\ +\overset{R_{1}}{\underset{r_{l}=1}{\sum }}CTTD{\left({\left\{T\right\}}_{t=1}^{m}\right)}_{:,r_{1},r_{m+1}}*\left(U_{:,r_{1}}^{1}+V_{:,r_{1}}^{1}\right) \end{array}$$

where,


(8a) $$\begin{array}{l} \overset{R_{1}}{\underset{r_{l}=1}{\sum }}CTTD{\left({\left\{T\right\}}_{t=1}^{m}\right)}_{:,r_{1},r_{m+1}}*\left(U_{:,r_{1}}^{1}+V_{:,r_{1}}^{1}\right) \end{array}$$

(8b) $$\begin{array}{l} =\overset{R_{1}}{\underset{r_{1}=1}{\sum }}\left(\overset{R_{2}}{\underset{r_{2}=1}{\sum }}\cdots \overset{R_{m}}{\underset{r_{m}=1}{\sum }}T_{:,r_{1},r_{2}}^{1}*\cdots *T_{:,r_{m},r_{m}+1}^{m}\right)\\ {}*\left(U_{:,r_{1}}^{1}+V_{:,r_{1}}^{1}\right) \end{array}$$

(8c) $$\begin{array}{l} =\overset{R_{2}}{\underset{r_{2}=1}{\sum }}\left(\overset{R_{3}}{\underset{r_{3}=1}{\sum }}\cdots \overset{R_{m}}{\underset{r_{m}=1}{\sum }}T_{:,r_{2},r_{3}}^{1}*\cdots *T_{:,r_{m},r_{m}+1}^{m}\right)\\ {}*\left(\overset{R_{m}}{\underset{r_{m}=1}{\sum }}T_{:,r_{1},r_{2}}^{1}*\left(U_{:,r_{1}}^{1}+V_{:,r_{1}}^{1}\right)\right) \end{array}$$

(8d) $$\begin{array}{l} =\overset{R_{2}}{\underset{r_{2}=1}{\sum }}CTTD{\left({\left\{T\right\}}_{l=2}^{m-1}\right)}_{:,r_{2},r_{m+1}}*V_{:,r_{2}}^{2} \end{array}$$

and by plugging Equation (8d) into Equation (7c), we reduce Equation (7c) back to the form of Equation (6), which completes the induction:


(9) $$\begin{array}{l} V_{:,r_{m}+1}=\overset{m}{\underset{l=2}{\sum }}\overset{R_{l}}{\underset{r_{l}=1}{\sum }}CTTD{\left({\left\{T\right\}}_{t=l}^{m}\right)}_{:,r_{l},r_{m+1}}\\ \;*\;U_{:,r_{l}}^{l}+\overset{R_{2}}{\underset{r_{2}=1}{\sum }}CTTD{\left({\left\{T\right\}}_{l=2}^{m-1}\right)}_{:,r_{2},r_{m+1}}*V_{:,r_{2}}^{2} \end{array}$$

### 5.3. Physics Constraints

In this section, we provide how the law of physics that describes the physical processes (e.g., the LCS) can be incorporated into the deep learning framework.

#### 5.3.1. Physics Equations

Physical processes are often modeled by a set of nonlinear partial differential equations (PDEs), which describe how a physical quantity is changed in a given region over time. Such dynamic equations are usually written as a relation between time derivatives and spatial derivatives such as:


(10) $$\begin{array}{l} \overset{P}{\underset{i=1}{\sum }}b_{i}\left(u,t\right)\frac{\partial^{i}u}{\partial t^{i}}=\overset{Q}{\underset{i=1}{\sum }}c_{i}\left(u,x\right)\frac{\partial^{i}u}{\partial x^{i}} \end{array}$$

where *u* is a physical quantity, say, velocity, and *x* is its spatial coordinate. Furthermore, coefficients *b*_*i*_, *c*_*i*_ are functions of *u* and *x*. Finally, *P* and *Q* denote the highest order of time derivatives and spatial derivatives, respectively.

There are multiple ways to incorporate the law of physics into deep neural networks. In this paper, inspired by Seo and Liu (2019), we modeled the law of physics by replacing the updating functions in neural networks with corresponding operators. Considering the characteristics of ocean current, we adopt two dynamic equations, i.e., the diffusion equation and the wave equation, shown in Table 2. The diffusion equation describes the behavior of continuous physical quantities (macroscopic behavior of many micro-particles in Brownian motion or turbulence in oceanic flows) resulting from the random movement. The wave equation is a second-order PDE for the description of waves (e.g., water, sound, or seismic waves), which are present in the current signal of the LCS through the propagation of wide range of perturbations (Donohue et al., 2016a,b).

**Table 2:** Ocean current related dynamic equations.

**Updating function**	**Dynamic equation**
$v_{i}^{\prime }=v_{i}+\alpha {\left(△v\right)}_{i}$	Diffusion: $\dot{u}=\alpha {▽}^{2}u$
$v_{i}^{\prime \prime }=2v_{i}^{\prime }-v_{i}+c^{2}{\left(△v^{\prime }\right)}_{i}$	Wave: ü = *c*^2^▽^2^*u*

As an illustrative example, let us consider time derivatives of the following liner wave equation:


(11) $$\begin{array}{l} \frac{\partial^{2}u\left(x,t\right)}{\partial t^{2}}=c^{2}\frac{\partial^{2}u\left(x,t\right)}{\partial x^{2}} \end{array}$$

A discrete analog of this equation is usually considered in the form of a second order finite difference equation, which is consistent with the updating function $v_{i}^{\prime \prime }-2v_{i}^{\prime }+v_{i}$ in Table 2. In the continualization method (Tarasov, 2017), it is assumed that the continuous displacement *u*(*x, t*) equals the lattice displacement *u*_*n*_(*t*) at particle *n* by *u*_*n*_(*t*) = *u*(*nh, t*), where the particle spacing is denoted by *h*. Thus, Equation (11) can then be written as:


(12) $$\begin{array}{l} \frac{\partial^{2}u_{n}\left(t\right)}{\partial t^{2}}=\frac{c^{2}}{h^{2}}\left(u_{n+1}\left(t\right)-2u_{n}\left(t\right)+u_{n-1}\left(t\right).\right) \end{array}$$

This can be shown as follows. Applying the Fourier series transform $\hat{f}\left(k\right)=\sum_{n=-\infty }^{+\infty }f\left[n\right]e^{-iknh}={𝔉}_{h,\Delta }\left\{f\left[n\right]\right\}$ to Equation (12) gives:


(13) $$\begin{array}{l} \frac{\partial^{2}{û}_{n}\left(k,t\right)}{\partial t^{2}}=-\frac{2c^{2}}{h^{2}}\overset{+\infty }{\underset{m=1}{\sum }}\frac{{\left(-1\right)}^{m}}{\left(2m\right)!}{\left(kh\right)}^{2m}û\left(k,t\right) \end{array}$$

and applying the inverse Fourier integral transform $f\left(x\right)=\frac{1}{2\pi }\int_{-\infty }^{+\infty }dk\hat{f}\left(k\right)e^{ikx}={𝔉}^{-1}\left\{\hat{f}\left(k\right)\right\}$ to the latter gives:


(14) $$\begin{array}{l} \frac{\partial^{2}u\left(x,t\right)}{\partial t^{2}}=\frac{2c^{2}}{h^{2}}\overset{+\infty }{\underset{m=1}{\sum }}\frac{{\left(h\right)}^{2m}}{\left(2m\right)!}\frac{\partial^{2m}u\left(x,t\right)}{\partial x^{2m}} \end{array}$$

Equation (14) becomes the wave Equation (11) in the limit *h* → 0, since,


(15) $$\begin{array}{l} \underset{h\to 0}{\lim}\frac{2}{h^{2}}\overset{+\infty }{\underset{m=1}{\sum }}\frac{{\left(h\right)}^{2m}}{\left(2m\right)!}\frac{\partial^{2m}u\left(x,t\right)}{\partial x^{2m}}=\frac{\partial^{2}u\left(x,t\right)}{\partial x^{2}} \end{array}$$

As a result, the discrete Equation (12) can be considered as an approximation of the wave Equation (11).

#### 5.3.2. Physics-Informed Learning

In Equation (3), a sliding window strategy was adopted. As shown in Figure 2, sliding subsets of $\left\{H^{l}\right\}$ were concatenated and then transformed into *m* (i.e., the number of core tensors in φ) intermediate hidden tensors $\left\{H^{\left(t,o\right)}\right\}$. The concrete process is as follows:


(16) $$\begin{array}{l} H^{\left(t,o\right)}=K^{o}*\left[H^{\left(t-n+m-l\right)};\cdots \;;H^{\left(t-l\right)}\right] \end{array}$$

where *n* is the sliding window size. The ${\left\{H\right\}}_{l=1}^{n}$ were first concatenated into *m* tensors ${\left\{H_{cat}\right\}}_{l=1}^{m}$ ($H_{cat}\subseteq {\mathbb{R}}^{H\times W\times \left(n-m+1\right)\times C}$) along the time axis, which were thereafter mapped to $H^{\left(t,o\right)}\subseteq {\mathbb{R}}^{H\times W\times R}$ by the 3D convolutional kernel $K^{o}\subseteq {\mathbb{R}}^{K\times K\times \left(n-m+1\right)\times R}$. Then, the intermediate hidden tensor $\left\{H^{\left(t,o\right)}\right\}$ was updated to $\left\{{\tilde{H}}^{\left(t,o\right)}\right\}$ with physics constraints.

It is hardly practical to simulate a complicated real-world system with the operators alone unless all physics equations governing the observed phenomena are explicitly known. To date, the LCS is not fully understood, making it almost infeasible to employ all related fluid equations to model the LC and its eddies. Consequently, it is necessary to utilize the learn-able parameters in neural networks to extract latent representations H in LC observations, which is then further incorporated with domain physics (i.e., constrained by physics equations). The physics-informed part within each PITT-ConvLSTM cell is shown in Algorithm 1.

**Algorithm 1:**
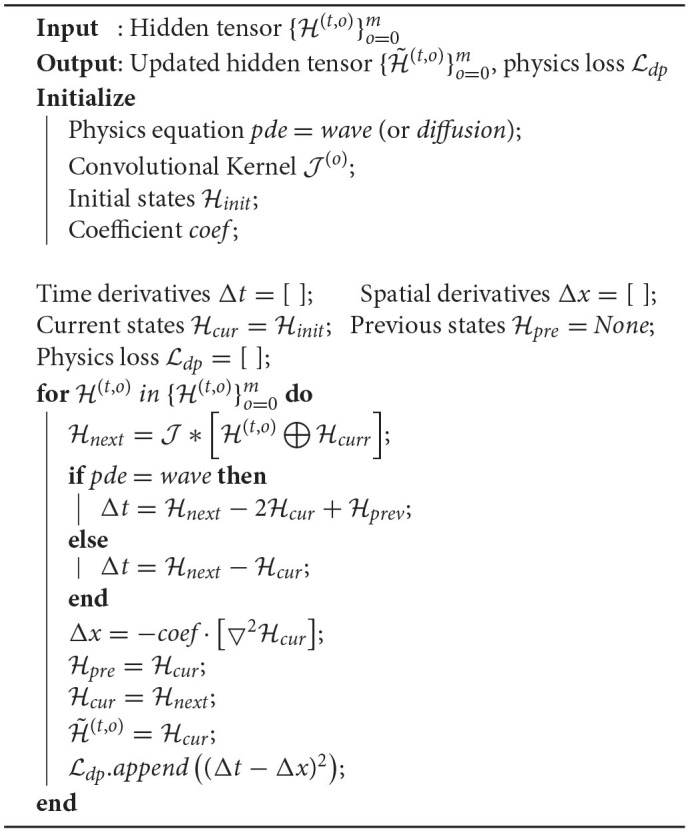
Intermediate Hidden Tensor Updating with Physics Constraints

Finally, the sequentially updated intermediate hidden tensors were transformed to the output space by convolutional tensor-train decomposition described in section 5.2. It is notable that the law of physics is not directly constrained by the raw observations, but rather by the latent representations (i.e., the hidden states of the ConvLSTM in this work). This is a desired configuration considering the difficulty in identifying which observations are following the law of physics explicitly and how much in accordance. Consequently, instead of individually applying the equation to each observation, we found that it is more efficient to introduce the constraints on the latent representations.

Specifically, as illustrated in Figure 2, J is the learn-able parameter vector while the orange blocks (“Physics Equations”) are objective functions related to physics constraints. First, we defined the physics constraints between the previous and updated states based on the known/assumed knowledge as:


(17) $$\begin{array}{l} L_{dp}^{\left(t,o\right)}=\text{g}\left(H^{\left(t,o\right)},{\tilde{H}}^{\left(t,o\right)}\right) \end{array}$$

where *g* is case-specific. In particular, if we are aware/assume that the observations should have a diffusive property, the diffusion equation can be used as the physics-informed constraint as:


(18) $$\begin{array}{l} {\text{g}}_{dif}={\left\|v^{\prime }-v-\alpha {▽}^{2}v\right\|}^{2} \end{array}$$

where *v* (or *v*′) is the corresponding element of H (or $\tilde{H}$). Finally, the physics-informed objective function of the total sequence is defined as:


(19) $$\begin{array}{l} L_{dp}=\overset{T}{\underset{t}{\sum }}\overset{m}{\underset{o=1}{\sum }}\text{g}\left(H^{\left(t,o\right)},{\tilde{H}}^{\left(t,o\right)}\right) \end{array}$$

and the overall objective function is the sum of the supervised loss and physical loss:


(20) $$\begin{array}{l} L=L_{1}+L_{2}+\lambda L_{dp} \end{array}$$

where $L_{1}$ is the mean absolute error, $L_{2}$ is the mean squared error, and λ controls the importance of the physics term.

## 6. Results and Discussion

### 6.1. Model Implementation

All experiments, shown in Figure 3, used a stack of 4 blocks (3 stacked layers of TT-ConvLSTM or PITT-ConvLSTM per block) and two skip connections added between the 1st and 3rd, 2rd and 4th blocks that perform concatenation over channels (Byeon et al., 2018). The channels are set to 32 for the 1st and 4th blocks and 48 for the middle blocks. A convolutional layer is applied on top of all the recurrent layers to compute the predicted frames. The PITT-ConvLSTM is of order 3, sliding window step size 3, rank 8 (the hyper-parameter tuning is based on the validation dataset. we considered an order of the decomposition from [1, 2, 3, 5], tensor ranks from [4, 8, 16] and number of hidden states in sliding window from [1, 3, 5] Su et al., 2020). All models were trained with the ADAM optimizer (Kingma and Ba, 2014) with objective function in Equation (20). Once the model did not improve after 20 epochs (in terms of validation loss), scheduled sampling (Bengio et al., 2015) was then activated to ease the training with linearly decreased sampling ratio from 1 to 0. Learning rate decay was further activated if the loss did not drop after 20 epochs, and the rate was decreased exponentially by 0.98 every 5 epochs. The initial learning rate is set to 10^−4^. All models were trained to predict 10, 20, 30 frames given 10 input frames, where we use the teacher forcing mechanism (Su et al., 2020; Zhuang and Ibrahim, 2021), when a step is within the number of input steps, it's direct multi-step forecast strategy, when the step exceeds the number of input steps, it adopts recursive multi-step forecast strategy. These baseline models are listed below:

1. ConvLSTM is first proposed by Xingjian et al. (2015) for precipitation nowcasting by extending the fully connected LSTM (FC-LSTM) to have convolutional structures in both the input-to-state and state-to-state transitions. The spatio-temporal information is encoded in each cell.
2. PredRNN proposed in Wang et al. (2017), is a recurrent network for spatiotemporal predictive learning. The core of PredRNN is a new Spatio-temporal LSTM unit that extracts and memorizes spatial and temporal representations simultaneously.
3. TT-ConvLSTM in Su et al. (2020) is a higher-order generalization to ConvLSTM, which is able to learn long-term spatio-temporal structure in videos.

### 6.2. Results

Two metrics were adopted to evaluate the performance and provide frame-wise quantitative comparisons. Specifically, the mean squared error (MSE) was used for element-wise differences, while the structural similarity index measure (SSIM) (Wang et al., 2004), which ranges between –1 and 1, was used for perceptual similarity. Here, we report the averaged MSE and SSIM over 10, 20, 30 predict horizons.

**Figure 5:**
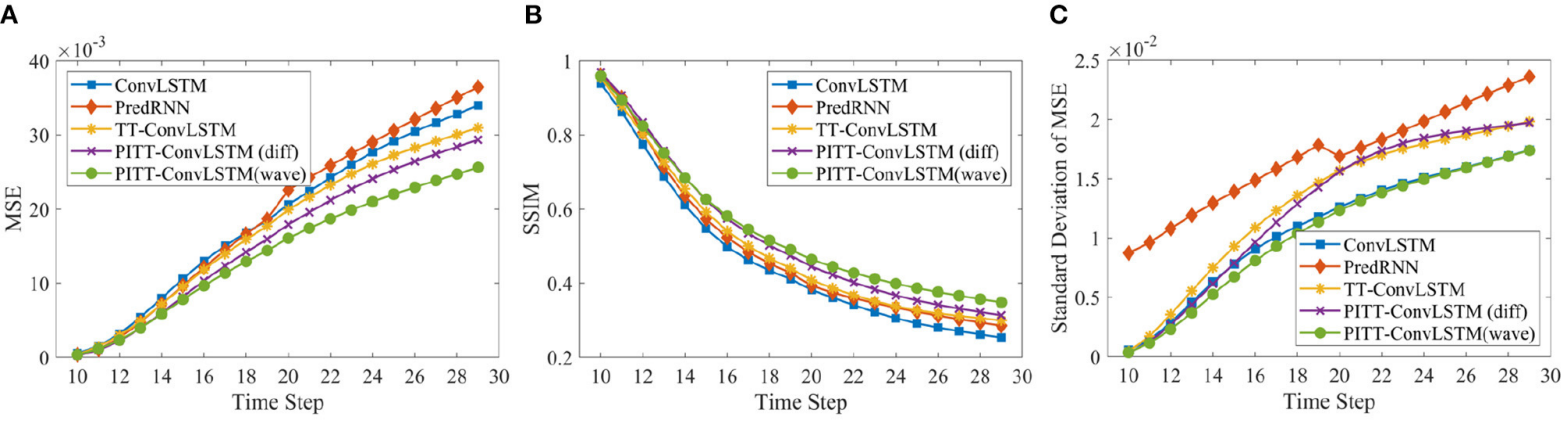
Frame-wise comparison in MSE (×*m*.*s*^−1^ × 10^−3^), Standard deviation of MSE (×*m*.*s*^−1^ × 10^−2^), and SSIM.

The average evaluation statistics, i.e., MSE and SSIM scores, over 10, 20, and 30 frames prediction is given in Table 3. Figure 5 shows the comparisons of the predictions by ConvLSTM, PredRNN, TT-ConvLSTM and proposed PITT-ConvLSTM informed by *wave* and *diffusion* equations. The ConvLSTM model generates blurred future frames (lowest SSIM), since it fails to memorize the detailed spatial representations. The reason lies in that the ConvLSTM is a first-order Morkovian model in nature, which means the hidden state is updated based on its previous step. Thus, it can not efficiently capture higher-order temporal correlations. ConvLSTM is more effective in short-term forecasting, focusing on next or first few frames prediction. By contrast, as shown in Table 3 the methods using convolutional tensor-train decomposition, TT-ConvLSTM and PITT-ConvLSTM, achieve better performance in long-term prediction with fewer parameters.

**Table 3:** Comparisons of 10, 20, and 30 time-steps prediction (equal to 5, 10, and 15 days ahead forecast), where lower MSE (in 10^−3^ magnitude) or higher SSIM indicates better model performance.

**Methods**	**10 → 10**		**10 → 20**		**10 → 30**		**Parms**
	**MSE**	**SSIM**	**MSE**	**SSIM**	**MSE**	**SSIM**	
ConvLSTM (Xingjian et al., 2015)	9.257	0.623	18.584	0.465	26.725	0.374	4.94 m
PredRNN (Wang et al., 2017)	8.752	0.648	19.229	0.490	27.512	0.351	8.84 m
TT-ConvLSTM (Su et al., 2020)	8.575	0.656	17.445	0.498	23.802	0.420	**0.97** m
PITT-ConvLSTM (diffusion)	7.429	0.686	15.835	0.527	22.426	0.439	0.99 m
PITT-ConvLSTM (wave)	**6.971**	**0.687**	**14.097**	**0.543**	**19.638**	**0.463**	0.99 m

**Figure 6:**
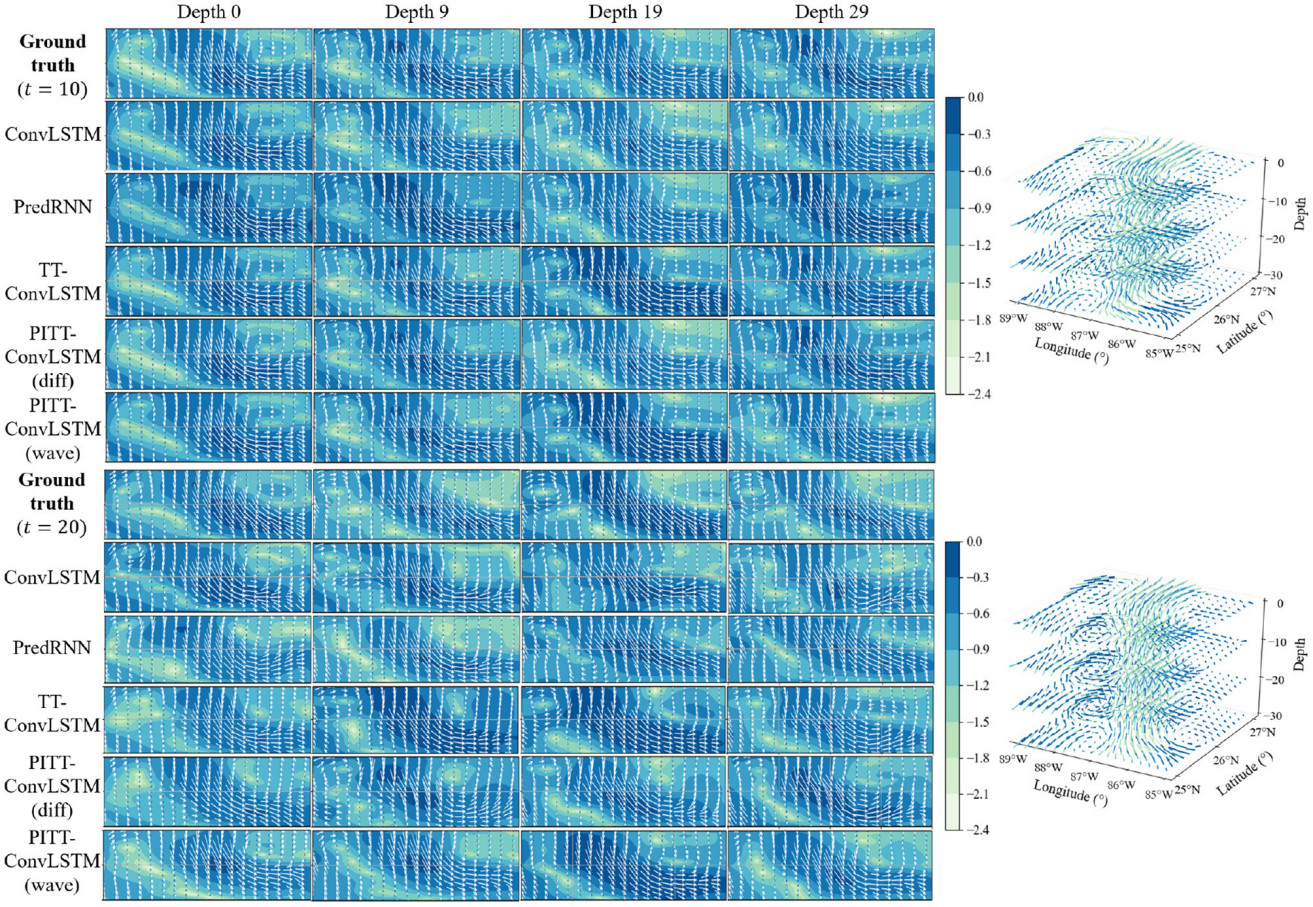
Left: The visualization of LCS prediction in 2D space. Every 10 (×12 h) steps ahead prediction with every 10 (×20 m) depth slices for each model are illustrated. The color map indicates log-based LC velocity magnitude, i.e., $\mathrm{lg}\sqrt{u^{2}+v^{2}}$. Right: The visualization of ground truth LCS in 3D space.

The proposed PITT-ConvLSTM models showed superior skills for both measures over the other models. It validates that the convolutional tensor-train, combined with physics constraints, is capable of modeling higher-order spatio-temporal correlations. The results showed how the physics constraints helped improve the volumetric velocity prediction of the LCS. Among the convolutional tensor-train decomposition (CCTD) based models, PITT-ConvLSTM realized the smallest MSEs and highest SSIMs. It validates the effectiveness of incorporating physical rules in latent representation learning, since knowing physics-constrained neighboring information is helpful to infer its own states. Specifically, the wave-informed PITT-ConvLSTM consistently outperformed all baseline models and showed a superior predicting capability both spatially and temporally. The LC prediction comparison among ConvLSTM, PredRNN, and TT-ConvLSTM is visualized in Figure 6.

## 7. Conclusion

Understanding the dynamics of the LCS is fundamental to understanding the Gulf of Mexico's full oceanographic system, and vice versa. Hurricane intensity, offshore safety, oil spill response, fisheries, and the Gulf Coast economy are all affected by the position, strength, and structure of the LC and associated eddies. The LC's position varies greatly from its retracted state in the Yucatan Channel, directly east of the Florida Straits, to its extended state into the far northern and western Gulf. Of particular interest to the modeling community is the prediction of the velocity field and the duration of the LC circulation at a given location.

In this paper, we proposed a physics-informed tensor-train ConvLSTM that is capable of effectively capturing long-term spatiotemporal correlations in a temporal sequence of volumetric data. Within the PITT-ConvLSTM cell, a large convolutional kernel was factorized into a set of smaller core tensors through convolutional tensor-train decomposition. In the learnt latent space, physical domain knowledge in the form of PDEs over time and space were incorporated to facilitate learning. The performance of our proposed PITT-ConvLSTM model was demonstrated on volumetric velocity forecasting of the LCS in the GoM, and the results verified that our model can produce superior results compared to published state-of-the-art deep learning models, including ConvLSTM, PredRNN, and TT-ConvLSTM. The results of this study suggest that it is now possible to create reliable current forecasts for a period longer than 5 days. The MSE of the PITT-ConvLSTM was less than 0.03(*m*.*s*^−1^) 15 days ahead, which is a significant improvement over the current state of the art ocean numerical models (Cooper et al., 2016). Note that although the proposed model is a sequence-to-sequence prediction, a sequence of arbitrary length can be generated in a recursive manner. In a future study, we aim to design an effective prediction model that can capture simultaneously both fast changing local patterns and slow varying global trends. Furthermore, as suggested in Zhang et al. (2018), multiple complex factors should be considered or integrated in modeling, including spatial correlation (nearby and distant), various types of temporal correlation (closeness, period, trend), and external conditions (e.g., weather).

## Data Availability Statement

The original contributions presented in the study are included in the article/supplementary material, further inquiries can be directed to the corresponding author/s.

## Author Contributions

YH, HZ, and YT contributed to the conception and design of the study. JV organized the database. YH performed the statistical analysis and wrote the first draft of the manuscript. YH, YT, HZ, and LC wrote sections of the manuscript. All authors contributed to manuscript revision, read, and approved the submitted version.

## Funding

This work was partially supported by an Understanding Gulf Ocean Systems Phase II grant (#NAS/GRP 2000011052) from the National Academies of Sciences, Engineering, and Medicine (NASEM), and by an MRI grant (#1828181) from National Science Foundation (NSF).

## Conflict of Interest

The authors declare that the research was conducted in the absence of any commercial or financial relationships that could be construed as a potential conflict of interest.

## Publisher's Note

All claims expressed in this article are solely those of the authors and do not necessarily represent those of their affiliated organizations, or those of the publisher, the editors and the reviewers. Any product that may be evaluated in this article, or claim that may be made by its manufacturer, is not guaranteed or endorsed by the publisher.
